# Accouchement gémellaire différé: à propos de deux cas observés à la maternité du Centre Hospitalier de Creil

**DOI:** 10.11604/pamj.2014.19.104.4617

**Published:** 2014-09-29

**Authors:** Claude Cyrille Noa Ndoua, Meyssam Fattouh, Shamsa Mirdat, Jean Dupont Kemfang, Jean Marie Kasia, Christophe Di Pace

**Affiliations:** 1 Centre Hospitalier de Creil, Creil, France; 2 Faculté de Médecine et des Sciences Biomédicales de l'Université de Yaoundé I, Yaoundé, Cameroun; 3 Centre Hospitalier de Recherche et d'Application de la Chirurgie Endoscopique et de la Reproduction Humaine, Cameroun

**Keywords:** Accouchement gémellaire différé, tocolyse, cerclage, prématurité, Deferred twin birth, tocolysis, cerclage, prematurity

## Abstract

L'accouchement gémellaire différé définit un accouchement en deux ou plusieurs temps, avec l'expulsion spontanée d'un premier fœtus au deuxième ou au troisième trimestre, et un prolongement de la grossesse pour obtenir un accouchement du ou des fœtus restants en gestation le plus proche possible du terme. Cette technique est mise en œuvre, en cas de grossesse gémellaire pour prévenir la prématurité du fœtus restant après l'expulsion très prématurée d'un premier fœtus. Nous rapportons deux cas observés à la maternité du Centre Hospitalier de Creil avec des latences respectives de 3 et 52 jours pour lesquels nous discutons la prise en charge.

## Introduction

L'accouchement gémellaire différé constitue un véritable challenge en pratique obstétricale. En effet, il est question de prolonger la vie intra-utérine du ou des fœtus restants après l'expulsion trop prématurée du premier jumeau. Au regard du risque hémorragique, de l'infection et de la terminaison spontanée de la grossesse, le principal défi pour l’équipe obstétricale est d'envisager cette pratique. Ensuite, il est question de poser l'indication de celle-ci pour enfin définir les différentes actions à mener en fonction des spécificités de chaque cas.

## Patient et observation

### Premier cas

Mme M.G. âgée de 29 ans, G3P1 dont une IVG par curetage deux ans plus tôt. Elle était porteuse de l'antigène Hbs avec moins de 2000 copies. L'antigène Hbe était négatif. Elle avait été admise après expulsion du premier jumeau à domicile sur une grossesse gémellaire bichoriale biamniotique de 18 semaines et 3 jours. Elle ne contractait pas, le saignement était minime. A l'admission, les battements cardiaques du deuxième jumeau étaient réguliers, le col était dilaté à 4 cm et la poche des eaux était intacte. Le cordon avait été sectionné sous valve au ras du col. La patiente avait été hospitalisée et mise sous Amoxicilline+acide clavulanique 1gramme/ 8 heures. Les éléments de surveillance étaient: la CRP (C Reactive Protein) tous les jours, le PV (Prélèvement Vaginal) chaque semaine, 2 échographies par semaine, le saignement et les contractions utérines. Au troisième Hospitalisation, le col mesurait 50 mm. Il n'y avait pas de contractions. L'antibioprophylaxie avait été arrêté au 10e jour. Un cerclage prophylactique avait été mis en place à 23 semaines et 6 jours pour raccourcissement cervical à 29mm et une tocolyse orale à la Nicardipine instituée. A 24 semaines, l’épreuve d'hyperglycémie provoquée par voie orale (HGPO) était normale. Le bilan infectieux était resté négatif. A 25 SA et 2 jours, la patiente rompait la poche des eaux et les contractions utérines efficaces s'installaient quelques minutes plus tard. La patiente avait été transférée au CHU d'Amiens. Elle y avait été mise sous Ceftriaxone pour infection vaginale polymicrobienne à E. coli, Streptocoque et Klebsiella. A 25 SA et 6 jours, l'expulsion de J2 survenait après un épisode de saignement important. Il pesait 700 grammes avec un Apgar de 5 puis 4 et décédait à J2 de vie pour sepsis sévère et prématurité. L'examen du placenta avait mis en évidence des lésions de chorioamniotite d’âges différents.

### Deuxième cas

Mme E. S âgée de 27 ans, 2^ème^ geste, sans enfant, célibataire, avait consulté pour douleurs abdomino-pelviennes à type de contractions utérines et perte des eaux sur grossesse gémellaire évolutive de 24 semaines 2 jours. Dans ses antécédents, elle avait fait une interruption volontaire de grossesse (IVG) par aspiration à 8 semaines, 5 ans plutôt. Le déroulement de la grossesse actuelle était marqué par la réalisation d'un cerclage d'urgence à 21 semaines au décours d'une hospitalisation de 10 jours. Le bilan infectieux était normal. Elle avait été mise sous Nicardipine en prophylaxie. A l'admission, L'examen clinique avait retrouvé un bon état général. Les paramètres hémodynamiques étaient stables. La hauteur utérine était de 30 cm. Le fil de cerclage était bien visible. Le col était dilaté à 2cm. La poche des eaux n’était pas rompue. La CRP faite en urgence était 7.7 mg/l. L'enregistrement tocographique confirmait l'existence d'une activité utérine. L’échographie obstétricale avait mis en évidence une grossesse gémellaire normalement évolutive avec protusion des membranes amniotiques à travers le col; J1 et J2 pesaient respectivement 704 et 659 grammes. La patiente a été mise sous Amoxicilline; une dose initiale de 2 grammes puis 1 gramme toutes les 8 heures. La décision de tocolyse au Tractocile et de maturation pulmonaire au Celestene a été prise. Six heures plus tard survenait l'expulsion de J1 mort-né pesant 710 gr sans anomalie morphologique apparente. Apres expulsion de J1, la décision de pratiquer un accouchement gémellaire différé a été prise sur la base de la régression de l'activité utérine, l'intégrité de la 2eme poche des eaux, l'absence de saignements ni de signe infectieux et la régularité des bruits du cœur fœtal (BDC) de J2. Le cordon a été ligaturé sous valve au ras du col et les protocoles de tocolyse, antibioprophylaxie et maturation pulmonaire ont été poursuivis. Au deuxième jour d'hospitalisation, il n'y avait pas de contractions le Tractocile a été arrêté et la patiente mise sous Loxen 1 mg/h. Le troisième jour d'hospitalisation, la patiente a présenté un saignement vaginal de moyenne abondance en caillot associé à des contractions utérines. L'expulsion de J2 est survenue 2 heures plus tard. Le poids de naissance était de 700grammes avec un APGAR de 4-6-6, taille=31cm et Périmètre crânien =22cm. L’évolution post-partum était sans particularités et la sortie autorisée 6 jours plus tard. Le nouveau-né a été pris en charge pour Maladie des membranes hyalines et dysplasie broncho-pulmonaire. Le séjour hospitalier avait duré 90 jours. A 12 mois, soit 9 mois d’âge corrigé, la petite fille pesait 6720 grammes avec un développement psychomoteur normal. L'EFR (épreuve fonctionnelle respiratoire) et L'IRM (imagerie par résonance magnétique) étaient normales étaient normales.

## Discussion

Plusieurs définitions de l'accouchement gémellaire différé sont proposées dans la littérature. Depaul [1] parle d'un accouchement en deux temps alors qu'ARIAS [2] le définit comme un accouchement gémellaire pour lequel le jumeau 1 est sorti au 2^ème^ trimestre et le jumeau 2 au troisième trimestre. Cristinelli [3] propose une définition globalisante en parlant d'un accouchement en deux ou plusieurs temps, avec l′expulsion spontanée d′un premier fœtus au deuxième ou au troisième trimestre, et un prolongement de la grossesse pour obtenir un accouchement du ou des fœtus restants en gestation le plus proche possible du terme. Il est important d'intégrer la notion du terme dans la définition en sachant que les expulsions de premiers jumeaux survenant au premier trimestre n'interviennent pas dans le processus d'accouchement gémellaire différé [3]. Par ailleurs, les accouchements de premiers jumeaux survenant au troisième trimestre posent le problème du bénéfice inhérent à une telle procédure tant il vrai que les progrès en réanimation néonatales assurent une survie certaine aux nouveau-nés de plus de 28 semaines. C'est pourquoi certains auteurs recommandent que la technique soit mise en œuvre avant la 28^ème^ semaine [4]. Dans ce cas, l'accouchement gémellaire différé serait défini comme une pratique obstétricale consistant à prolonger la vie in utero du ou des jumeaux restants après sortie du premier jumeau au deuxième trimestre. Dans les deux cas présentés, le premier jumeau est sorti à 18 et 24 semaines respectivement. Tout le bénéfice de la technique réside dans le prolongement in utero du 2^ème^ jumeau qui est généralement un grand prématuré. Il s'agit d'une situation obstétricale à laquelle les praticiens sont de plus en plus confrontés, à cause des progrès de la médecine de la reproduction et le nombre croissant de grossesses multiples. Depuis Carson qui décrivit le premier cas en 1880 [5], plusieurs cas isolés sont rapportés dans la littérature. L'incidence réelle n'est pas connue. Aux Etats-Unis en 2005, Oyelese [4] présente une cohorte de 4257 grossesses gémellaires dont 6,1% des jumeaux accouchés de façon différée avec un premier jumeau né entre 22 et 28 semaines.

Le challenge en salle de naissance c'est d'y penser et ensuite de décider du protocole de prise en charge car il est souvent difficile de décider de facto de la réalisation d'un accouchement gémellaire différé. Dans le premier cas la décision ne s’était pas imposée d'emblée à cause d'une sortie trop précoce du 1er jumeau (18 semaines). Les chances de mener le 2^ème^ jumeau à un âge de viabilité ou de le récupérer en cas d'expulsion semblaient réduites. La grossesse a été cependant menée jusqu′à 25 semaines et 6 jours. S'agissant de la latence, Fayad [6] dans son étude portant sur 35 cas, trouve que la sortie précoce du premier jumeau est associée à délai plus long pour la sortie du 2^ème^ jumeau. Ceci serait lié à l'effet délétère du degré de dilatation (qui est fonction de l’âge gestationnel du premier jumeau) sur la continence cervicale. D'autres facteurs sous-jacents tels que les béances cervico-isthmiques qui entrainent des fausses couches de fin du 2^ème^ trimestre [7] sont également à prendre en compte pour expliquer la réduction du délai de latence en cas de sortie tardive du 2^ème^ jumeau. Ce qui pourrait expliquer le délai assez court de trois jours que nous avons observé dans notre deuxième cas.

Selon Cristinelli [3], certains préalables sont nécessaires pour réaliser un accouchement gémellaire différé. La présence d′une chorioamniotite cliniquement évidente, d′un HRP, d′une souffrance fœtale contre-indiquent ce type de technique. La grossesse doit être pluri-amniotique, pluri-choriale. Dans les deux cas que nous avons rapportés, les grossesses étaient bi-amniotiques et bichoriales. Le bilan infectieux initial était négatif et l’échographie n'avait pas mis en évidence un HRP dans les deux cas. A ces préalables, il faudrait ajouter l'absence de saignement important après la sortie du 1er jumeau.

Ainsi après validation de tous ces préalables, plusieurs gestes peuvent être posés. Ces gestes qui font l'objet pour la plupart de controverses peuvent avoir un effet sur le délai entre les dates de sortie des jumeaux. Les intervalles varient selon les cas rapportés. Dans ce sens, nous rapportons deux cas qui s'opposent avec des délais respectifs de 52 et 3 jours; l'issue ayant été malheureusement fatale pour le premier cas. Palmara et all [8] rapportent un délai de 154 jours avec une première expulsion à 16 semaines et un accouchement d'un bébé vivant à 38 semaines. Dans la série de Roman [9] portant sur 19 patientes, le délai moyen était de 16 jours et 3 patientes soient 15.8% ont eu un délai de moins de 24 heures. Un cas rare de survie du premier jumeau sorti à 22 semaines 6jours a été décrit par Kaneko [10]. L'auteur a décrit un intervalle de temps de 9 jours avec issue néonatale favorable pour les deux jumeaux.

La ligature du cordon au ras du col: Elle limite le risque infectieux selon la plupart des auteurs [5, 6, 11–13]. Dans notre cas, nous avions effectué une ligature sous valve au ras du massif cervical dans les deux cas. Surico [13] qui préconise une ligature par endoloop rapporte deux cas avec issue materno-fœtale favorable. La mise en route d'une Tocolyse: elle peut être utilisée comme attitude préventive si l'activité utérine s'arrête spontanément après la sortie du premier jumeau [3]. Pour autant, certains auteurs pensent que si l′activité utérine est inexistante après l′expulsion, l′emploi systématique des tocolytiques n′est pas nécessaire [5]. La tocolyse à visée prophylactique est utilisée dans 60% des cas [14]. Concernant notre deuxième cas, nous avons mis en route une tocolyse thérapeutique à base de Tractocile dès l'admission de la patiente qui a été arrêté pendant l'expulsion de J1 et réinstitué juste après. Au deuxième jour, avec la maitrise de l'activité utérine, l'Atosiban a été remplacé par la Nicardipine. Dans le 1^er^ cas, la tocolyse prophylactique n'avait pas été instituée. Elle n'a été mise route qu'après le cerclage fait à 23 semaines 6 jours à cause d'un raccourcissement cervical à 29 mm retrouvé à l’échographie. Les bienfaits de la tocolyse systématique sont diversement appréciés dans la littérature, tout comme les molécules utilisées à cet effet. Porreco [12] propose une tocolyse systématique alors que Wittman [15] estime que la tocolyse doit être instituée si les contractions utérines persistent après la sortie de J1 car dans certains cas, l'activité utérine s'arrête spontanément. Une telle attitude se justifierait par le fait que les contractions peuvent révéler une infection et donc les prévenir pourrait retarder le diagnostic de celle-ci [16]. L’étude de Porreco publiée en 2000 portait sur une série complète de 24 cas [12] pour lesquels une tocolyse avait été mise en place systématiquement comprenant sulfate de magnésie, indométacine pendant 48 heures et de la nitroglycérine en intraveineuse. L'auteur a rapporté 7 cas d'endométrites et une phlébite septique.

La Corticothérapie: elle est quasi systématique car le ou les jumeaux restant étant souvent de grand prématuré [15]. Après la première expulsion, le cerclage est utilisé dans 46% des cas dans la littérature [14]. Le débat reste d'actualité quant à la réalisation d′un cerclage après l'expulsion du premier. La mise en place systématique d′un cerclage reste controversée à cause d'un risque de rupture des membranes et un risque infectieux non négligeables [5]. D′autres auteurs ne recommandent un cerclage qu′en cas de dilatation persistante du col [15] et recommandent dans ce cas la réalisation systématique d'une échographie endovaginale. Toutefois, Zhang et al. [11] estiment que la réalisation d'un cerclage après l'expulsion du premier fœtus est associée à un délai de sortie plus long sans entraîner une augmentation du risque infectieux. Cependant, l'existence d'un cerclage antérieur semble raccourcir l'intervalle de temps entre la sortie des deux jumeaux [6] comme nous l'avons constaté dans le deuxième cas rapporté. Dans le même ordre d'idées, Porreco et all [12] trouvent que dans le groupe des patientes cerclées en début de grossesse et dont le cerclage a été enlevé pour l'accouchement du premier fœtus, le délai entre les deux accouchements est plus court que celles du groupe sans cerclage. Ceci serait dû au fait que sur un col initialement pathologique et donc cerclé, la rétraction cervicale après la sortie du premier jumeau et nécessaire à la continence du deuxième jumeau est désuète. Il n'est cependant pas recommandé de recercler ces patientes [14] c'est pourquoi dans notre deuxième cas, nous n'avons pas jugé utile de refaire un cerclage. De plus la pratique d'un cerclage en cas d'accouchement gémellaire différé n'est pas courante dans le service.

La problématique de l'antibioprophylaxie reste non moins une préoccupation majeure. Elle est quasi systématique [14]; car l'ouverture du premier œuf et la sortie du premier jumeau exposent à un risque accru d'infection par contamination ascendante. Nos patientes avaient bénéficié d'une antibioprophylaxie systématique après l'expulsion du premier jumeau. Le bilan infectieux était resté normal chez la deuxième patiente tandis que la première avait développé une chorioamniotite découverte à 25 semaines et 6 jours. Le problème de l'antibioprophylaxie se pose moins en termes de molécule qu'en termes de durée du traitement. Il semble évident que dans notre premier cas, nous ne pouvions pas maintenir un traitement antibiotique pendant 7 semaines! Ainsi, Cristinelli [3] propose que lorsque le délai tend à se prolonger, l'antibiotique soit maintenu pendant 7 à 10 jours et réintroduit si les signes infectieux apparaissent. Seulement il peut etre parfois difficile de mettre en évidence assez précocement certaines infections peu parlantes comme ce fut le cas de la chorioamniotite frustre développée par notre première patiente et donc l'issue a sans doute été fatale pour le nouveau-né posant par là la question de la nécessité de différer l'accouchement pour le 2^ème^ jumeau. Cependant, L'issue favorable dans le 2^ème^ cas clinique nous amène à penser que cette approche devrait être intégrée le cas échéant dans la pratique quotidienne en salle de naissance.

## Conclusion

La pratique de l'accouchement gémellaire différé est en nette progression du fait de l'augmentation de l'incidence des grossesses gémellaires. Dans des situations de grande prématurité il peut permettre au(x) fœtus resté(s) d'améliorer leur maturation. Par ailleurs, s'il évite parfois la mort du deuxième jumeau, il permet rarement d’éviter la grande prématurité et ses risques de séquelles. Il n'existe pas de consensus sur les modalités de sa réalisation mais il doit être pratiqué au cas par cas en respectant des conditions précises pour limiter les risques maternels et fœtaux inhérents à cette pratique obstétricale.
